# Supplementary material for: Metal-free photoanodes for C–H functionalization
Source: Nat Commun. 2023 Nov 4;14:7104. doi: 10.1038/s41467-023-42851-w (PMC10625597; doi:10.1038/s41467-023-42851-w)
Supplement: Supplementary file 1 — Supplementary Information [file 41467_2023_42851_MOESM1_ESM.pdf]

Supplementary Information

Metal-free photoanodes for C-H functionalization

Junfang Zhang[1,2,3], Yuntao Zhu[1], Christian Njel[4], Yuxin Liu[1,2], Pietro Dallabernardina[1], Molly M. Stevens[3], Peter H. Seeberger[1,2], Oleksandr Savateev[1,5], and Felix F. Loeffler[1]**

[1]Max Planck Institute of Colloids and Interfaces, Am Muehlenberg 1, 14476 Potsdam, Germany

[2]Department of Chemistry and Biochemistry, Freie Universität Berlin, Arnimallee 22, 14195 Berlin, Germany

[3]Department of Materials, Department of Bioengineering, and Institute of Biomedical Engineering, Imperial College London, London SW7 2AZ, United Kingdom

[4]Institute for Applied Materials (IAM) and Karlsruhe Nano Micro Facility (KNMFi), Karlsruhe Institute of Technology (KIT), Hermann-von-Helmholtz-Platz 1, 76344 Eggenstein-Leopoldshafen, Germany

[5]Department of Chemistry, The Chinese University of Hong Kong, Shatin, New Territories, Hong Kong, China

*E-mail: Oleksandr.Savatieiev@mpikg.mpg.de, Felix.Loeffler@mpikg.mpg.de

Keywords: carbon nitrides, film preparation, photoelectrochemistry, organic reactions, semiconductors

**Supplementary Information**	**Page**
**Fig. S1** Photoelectrode preparation	3
**Fig. S2** Surface roughness analysis	3
**Fig. S3** Photoelectrodes on different substrates	4
**Fig. S4** Photoelectrodes prepared with different concentration of polymer	5
**Fig. S5** CN vs DCN photoelectrodes before and after scratching the top layer	6
**Fig. S6** FT-IR spectra of the precursor-polymer mixture, CN, and DCN electrodes	7
**Fig. S7** ^{15}N solid-state NMR spectrum of DCN and CN films	8
**Fig. S8** UV-vis absorption spectra of DCN and blade-coated CN film	9
**Fig. S9** Steady-state fluorescence spectrum of a DCN electrode	9
**Fig. S10** Characterization of DCN/CN: Tauc, Mott-Schottky plots, energy diagram	10
**Fig. S11** Cyclic voltammogram of 10 mM Fc^+/Fc in 0.1 M $LiClO_4$	10
**Fig. S12** Stability measurement of DCN electrodes	11
**Fig. S13** Proposed mechanism of photoelectrochemical oxygenation of N-aryl THIQ	12
**Supplementary Discussion 1**	12
**Fig. S14** Proof of photoelectrocatalytic synthesis of 4-(4-methylphenyl)-morpholine	13
**Supplementary Discussion 2**	13
**Fig. S15** Experimental setup	14
**Fig. S16** ^{1}H NMR of the THIQ substrate and product	14
^{1}H NMR and ^{13}C NMR of compounds **2a-f**	15-20
**Table S1.** DCN trace metal analysis by inductively coupled plasma OES	21
**Supplementary Discussion 3**	21
**Table S2.** Current density of different DCN electrodes in water	22
**Table S3.** Current density of different DCN electrodes in methanol	22
**References**	23

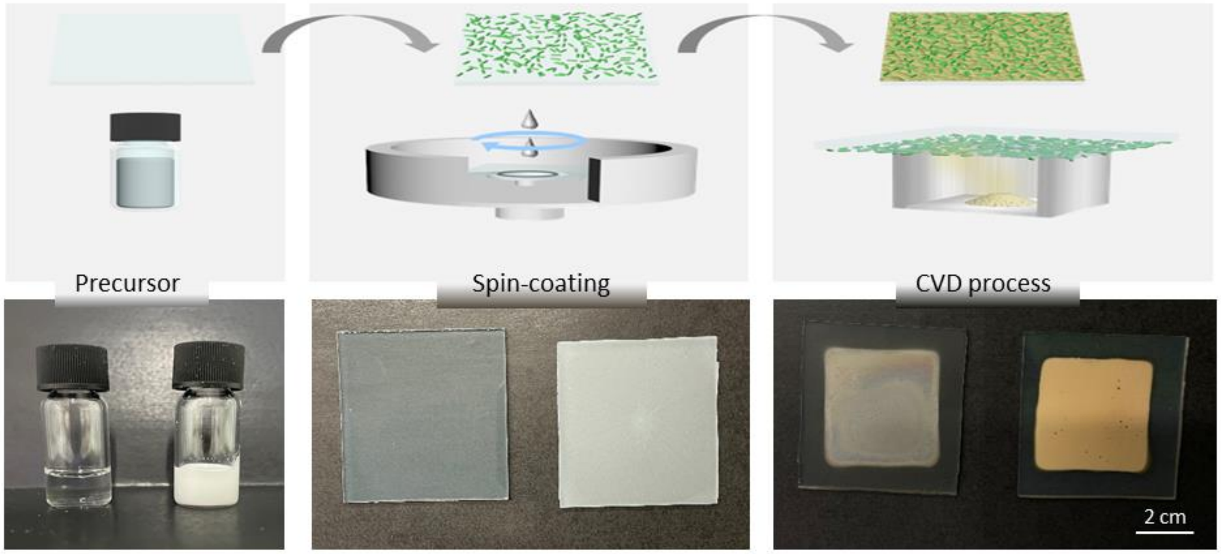

Fig. S1 Photoelectrode preparation through a spin-coating pre-treatment and CVD process. In the pre-treatment step, 50 mg melamine-cyanuric acid (MCA) supramolecular mixture and 50 mg polymer were dissolved or dispersed in 500 µL solvent. The prepared solution was spin-coated onto the FTO substrate to get a homogeneous precursor-polymer composite film, which will form the porous top layer in the dual-layer structure later. Specifically, S-LEC and PS are dissolved in DCM, while PVP and PEG are dissolved in water. Then, 5 g melamine were placed in a 29.5 mL rectangular alumina crucible. The FTO glass with the supramolecules and polymer complex films is placed on the top of the crucible. During the CVD process, the polymer in the composite film decomposed, resulting in the porous structure of the top layer. Melamine vapor passes through the top layer and condenses and polymerizes on the surface of the substrate, which gives the bottom layer in the dual-layer structure.

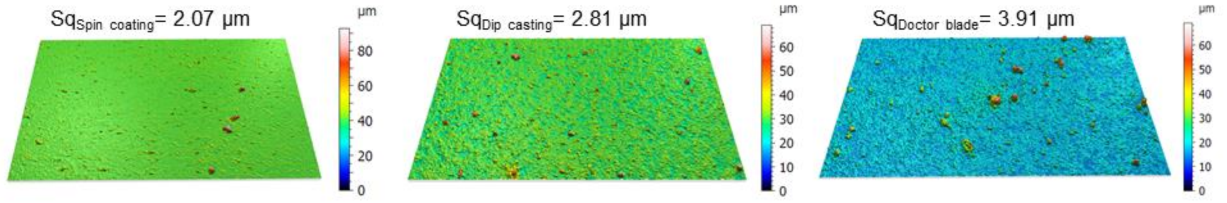

Fig. S2 Surface roughness analysis of the films prepared by spin coating, dip casting, and doctor blading. Sq is the Root-mean-square height, which is obtained by the following equation:

$$Sq = \sqrt{\frac{1}{A}\iint_A Z^2(x,y)dxdy}$$

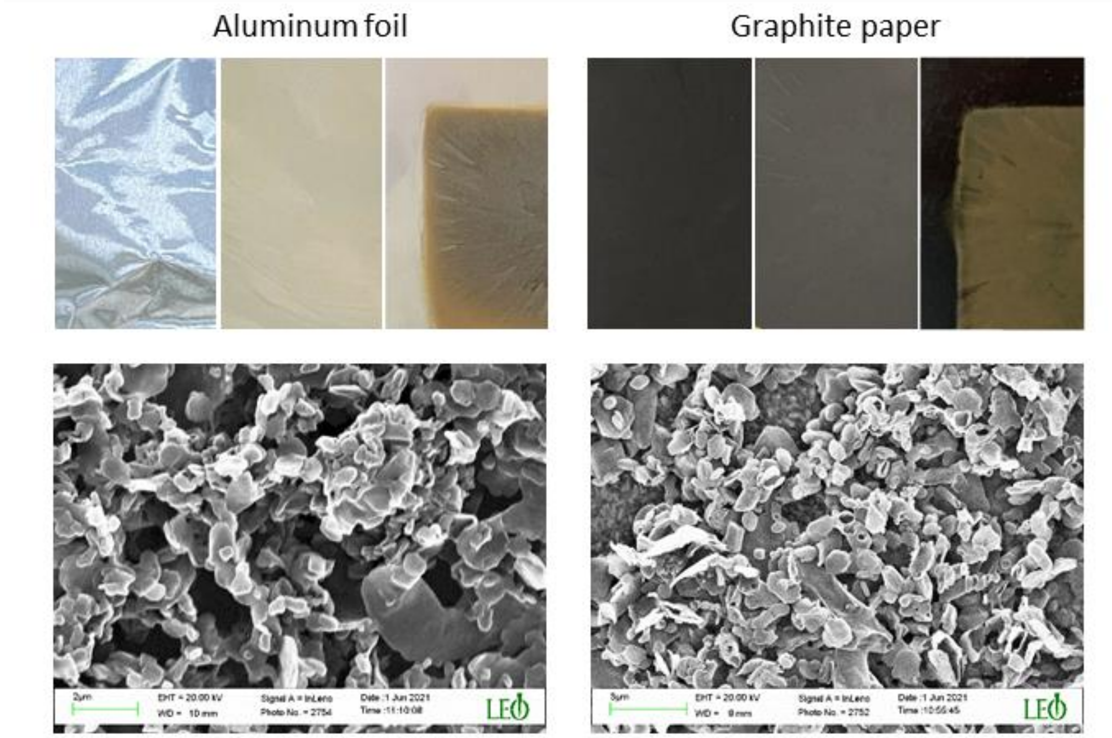

Fig. S3 Photoelectrodes on different substrates are able to be generated by our approach. For each substrate, the bare surface, the film after spin-coating pre-treatment, and the final electrodes with microstructures observed by SEM are shown.

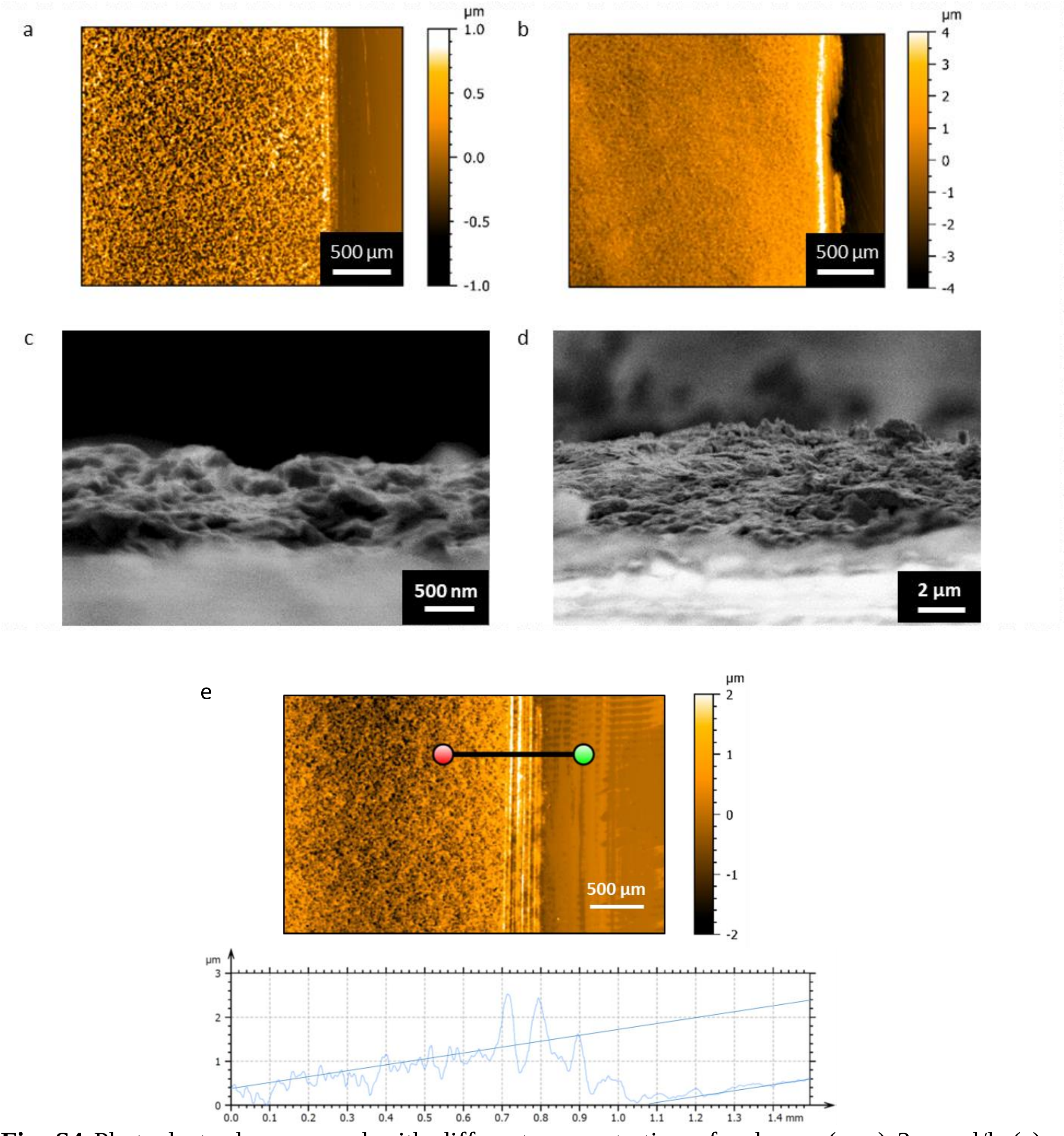

Fig. S4 Photoelectrodes prepared with different concentration of polymer (a, c) 3 mmol/l, (e) 6 mmol/l, (b, d) 12 mmol/l, and their topographies are measured by both white light interferometry and cross-section SEM, which give consistent results. (e) Topography and line profile of a photoelectrode with 6 mmol/l polymer, measured by white light interferometry. The film thickness is about 1.8 µm.

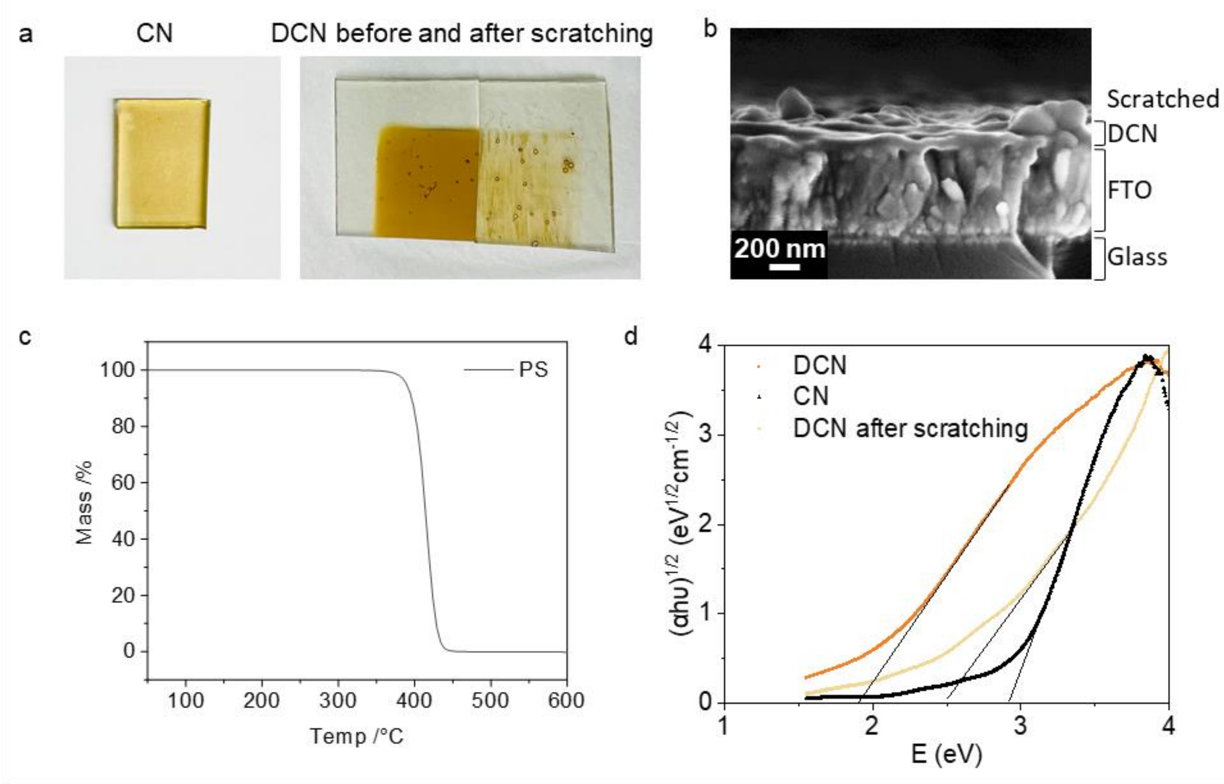

Fig. S5 CN vs DCN photoelectrodes before and after scratching the top layer. (a) CN photoelectrode prepared by CVD process without spin-coating pre-treatment and the DCN photoelectrodes before and after scratching the top layer. (b) Cross-section SEM image of DCN photoelectrode after scratching the top layer. (c) Thermogravimetric analysis (TGA) of polystyrene (PS). (d) Tauc plots of photoelectrodes.

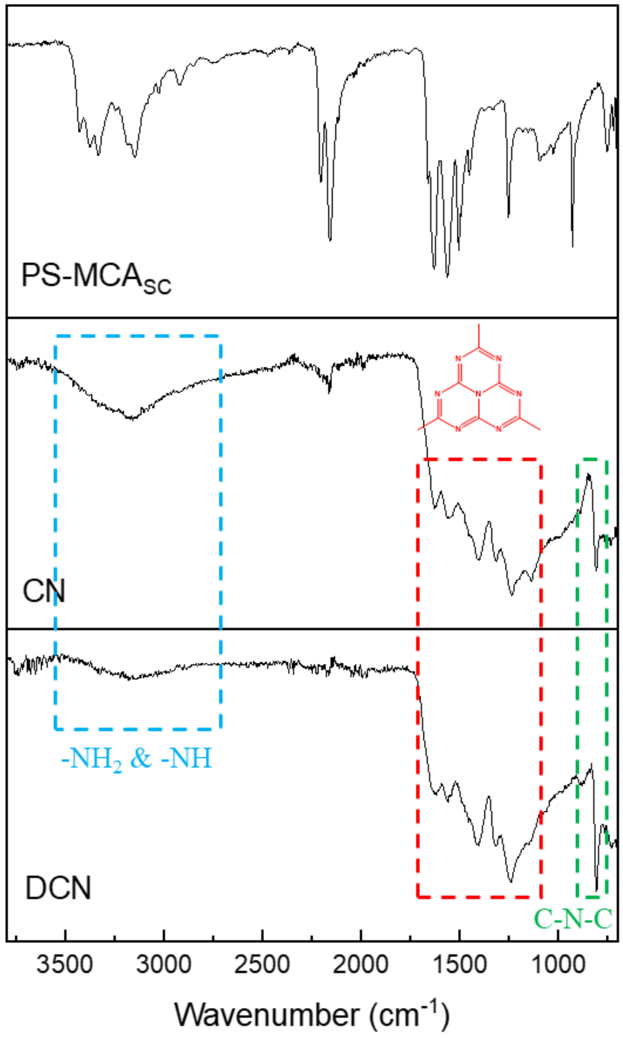

Fig. S6 Fourier transform infrared spectra of the precursor-polymer mixture, CN, and DCN electrodes.

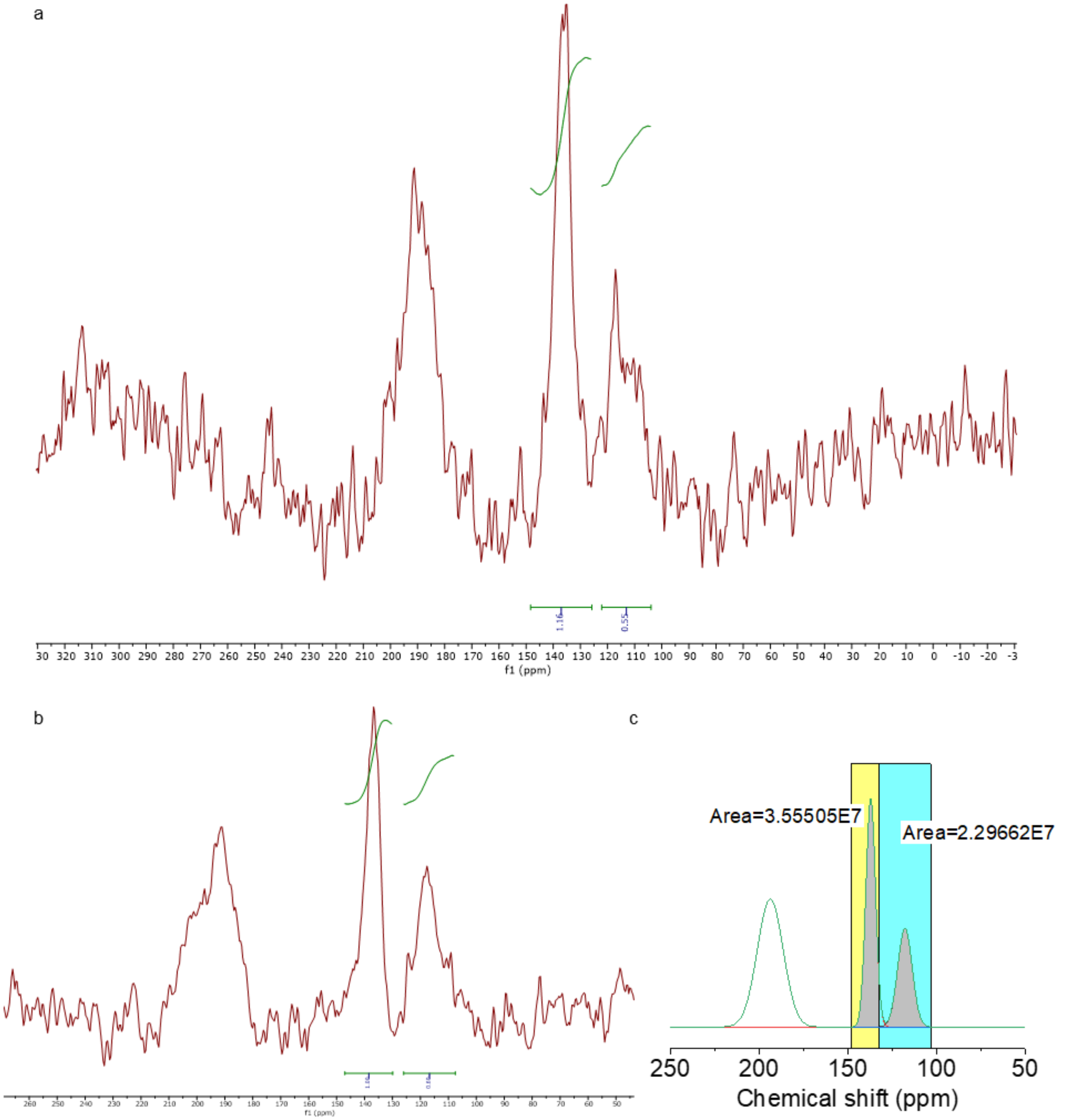

Fig. S7 ^{15}N solid-state NMR spectrum of (a) DCN films after devolution, CN films (b) before and (c) after devolution (Bruker 600, 8kHz, CP/MAS).

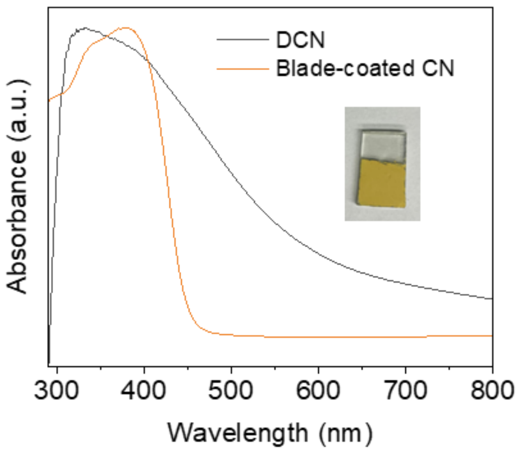

Fig. S8 UV-visible absorption spectra of DCN and blade-coated CN film (inset). After preparation of the DCN electrode by CVD, the remaining CN powder in the crucible was mixed with ethylene glycol (10 mg/ml). The mixture was ground and the suspension was deposited onto an FTO substrate by doctor blading (see inset). The film was dried on a hot plate at 70 °C, yielding a strongly scattering film. UV-vis absorption measurements were performed in transmittance mode using a PG Instruments TG70+ UV/vis spectrometer.

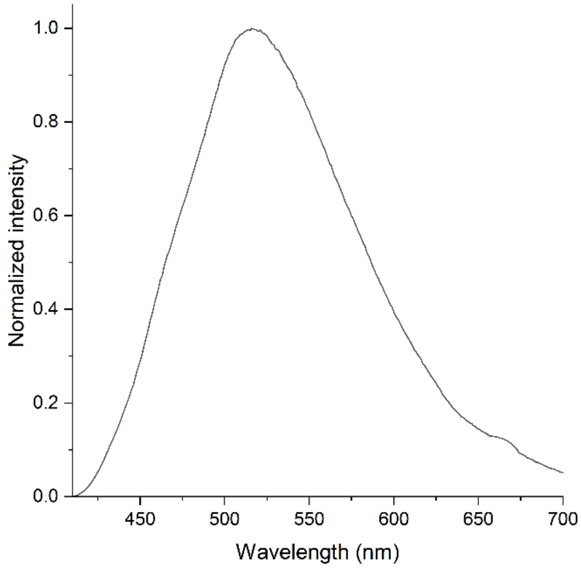

Fig. S9 Steady-state fluorescence spectrum of a DCN electrode acquired using a picosecond laser diode (excitation at 375 nm) in a PicoQuant TCSPC FluoTime 250 spectrometer operating at steady-state mode.

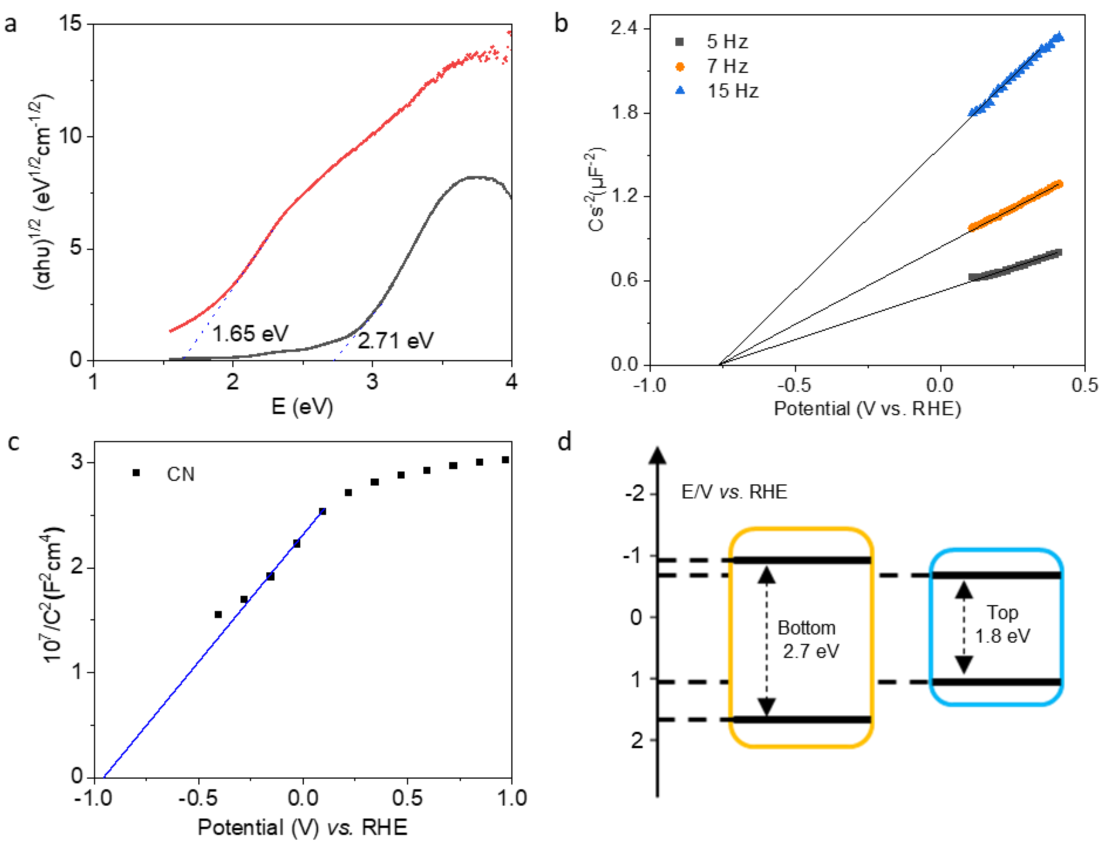

Fig. S10 Characterization of DCN vs CN electrodes. (a) Tauc plots of DCN (red) and CN (black). Mott-Schottky plots of (b) DCN and (c) CN.[1] (d) Energy diagram of DCN.

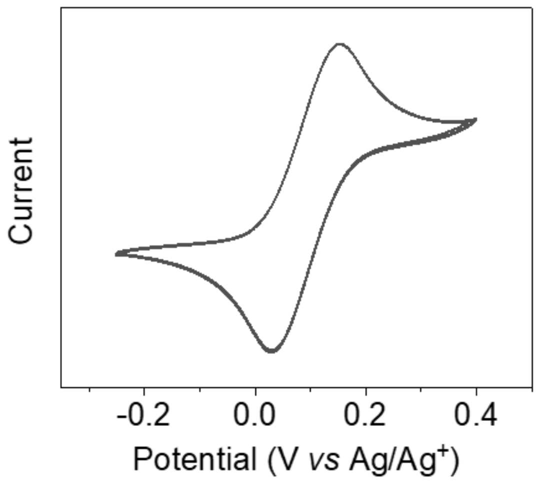

Fig. S11 Cyclic voltammogram of 10 mM Fc^+/Fc in 0.1 M $LiClO_4$ in methanol at a scan rate of 50 mV s^{-1}.

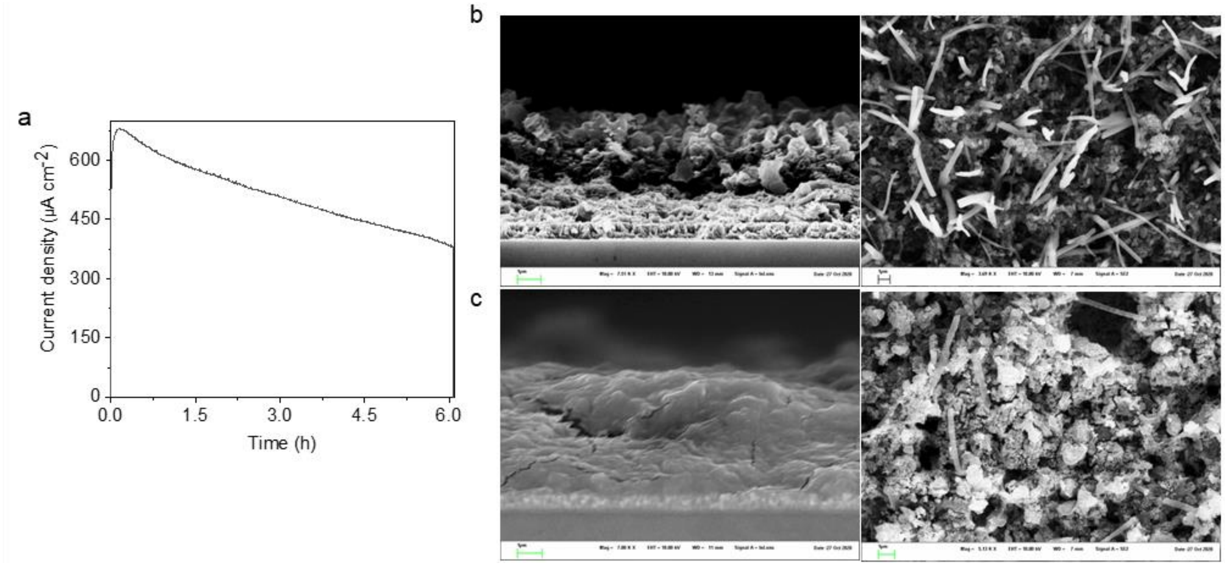

Fig. S12 The stability of DCN electrodes was measured under the conditions for PEC reactions (+0.22 V vs. Fc^+/Fc, 0.1 M $LiClO_4$ in methanol). SEM images of the DCN electrodes (b) before and (c) after the stability test.

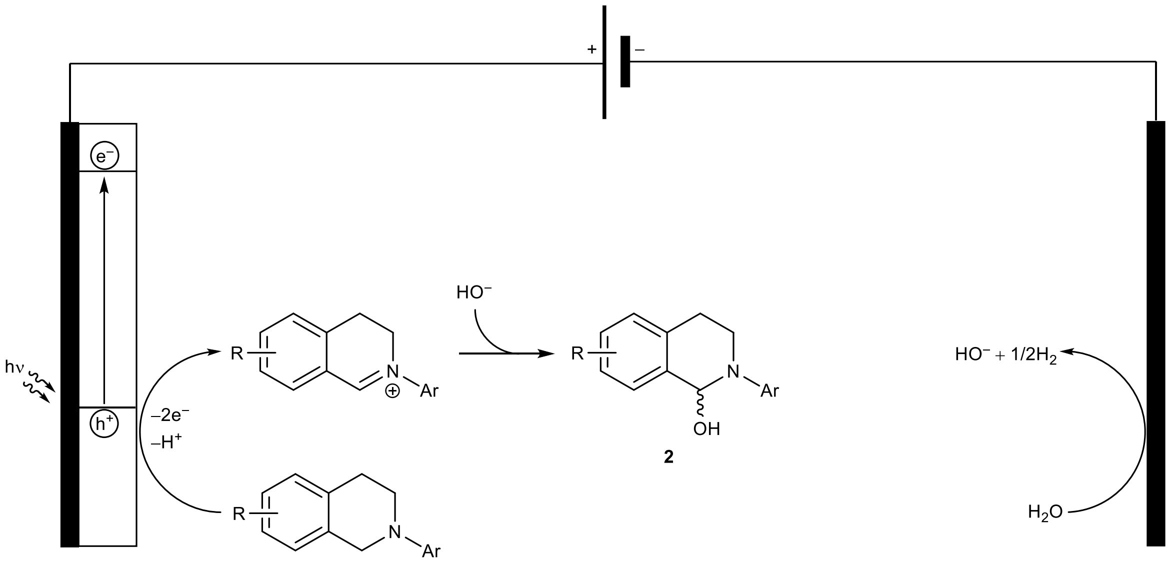

Fig. S13 Proposed mechanism of photoelectrochemical oxygenation of N-aryltetrahydroisoquinolines.

Supplementary Discussion 1

Excitation of the DCN photoanode by light converts carbon nitride into an excited state. The bias voltage of +0.22 V facilitates the separation of charges by extracting electrons. Photogenerated holes oxidize N-aryl-tetrahydroisoquinoline to the corresponding iminium cation ($2e^-/H^+$ process), which upon nucleophilic attack of HO^- is converted into the product. Iminium cations are ubiquitous intermediates in photoredox catalysis and were also postulated in photocatalytic functionalization of N-aryl-tetrahydroisoquinolines by carbon nitrides.[2, 3] Due to the constant bias potential of only +0.22 V vs. Fc+/Fc, compared to a constant current of 5 mA,[4] the aminoalcohol **2** is selectively obtained instead of the amides. HO^- species are replenished upon water reduction at the Pt cathode. Although the scope of the reaction was investigated in methanol, screening of reaction conditions revealed that the yield of 2a was high (86%), when the reaction was performed in wet acetonitrile or acetone (Table 1, entries 1,2). Compound **2** has been reported and characterized previously.[5]

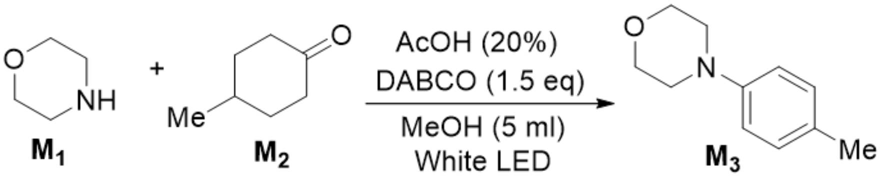

Entry	M_1/M_2	AcOH	DABCO	Potential	Time	Yield
1	1:1.2	50 μL	8.4 mg	0.58	12h	Trace
2	1:1.2	2 μL	8.4 mg	0.58	6h	Trace
3	1:1.2	2 μL	8.4 mg	0.22	6h	Trace
4	1:1.2	-	-	0.22	6h	-
5	1.2:1	100 μL	-	0.22	6h	-
6	1.2:1	1.5 μL	8.4 mg	0.22	4h	-

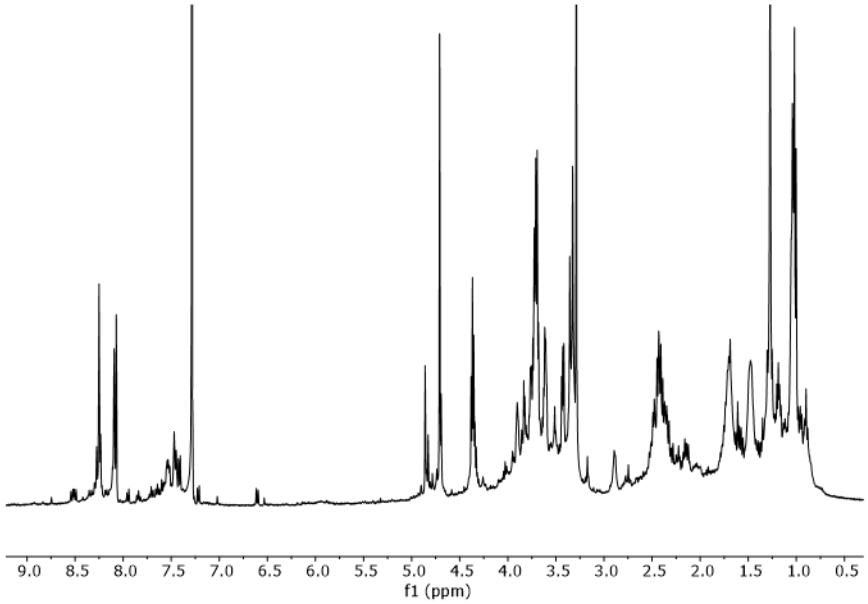

Fig. S14 Optimization of reaction conditions for the photoelectrocatalytic synthesis of 4-(4-methylphenyl)-morpholine.

Supplementary Discussion 2

To show the potential of our DCN photoelectrodes for other cross-coupling reactions, we investigated the synthesis of 4-(4-methylphenyl)-morpholine (Fig. S13).[6] A DCN photoelectrode, a Pt mesh, and $Ag/AgNO_3$ electrodes were used as working, counter, and reference electrodes, respectively, with 0.1 M $LiClO_4$ in methanol. Different conditions were investigated as shown in the table. After the reaction, the solution was diluted with 5 ml double-distilled H_2O and 5 ml ethyl acetate (EtOAc). After adding 1,3-dinitrobenzene (84 mg), separate layers could be observed. The aqueous layer was extracted twice with EtOAc (5 ml). The combined organic layers were washed with brine, dried with $MgSO_4$, filtered, and evaporated. Then, $CDCl_3$ (0.5 mL) was added for ^{1}H NMR analysis. After the reaction, peaks around 7.5 ppm appeared, indicating the formation of aromatic rings. Even though the yield was low, these preliminary results show the potential of the DCN photoelectrodes for more complex C-C couplings after comprehensive optimization.

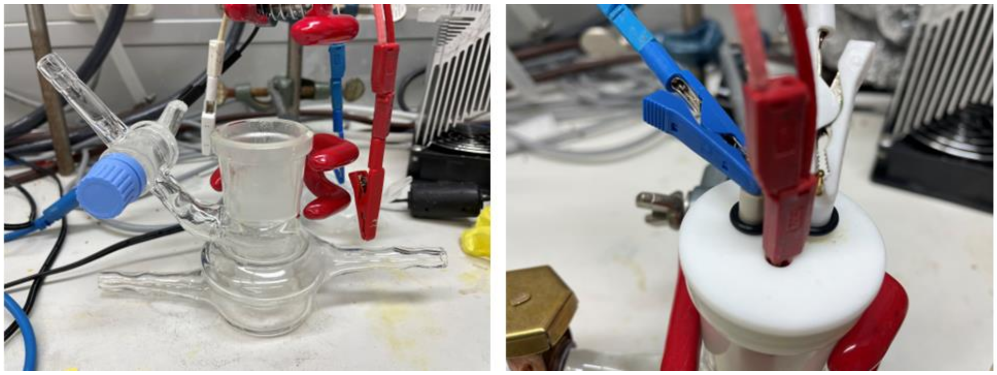

Fig. S15 Experimental setup for the photoelectrocatalytic C–H functionalization.

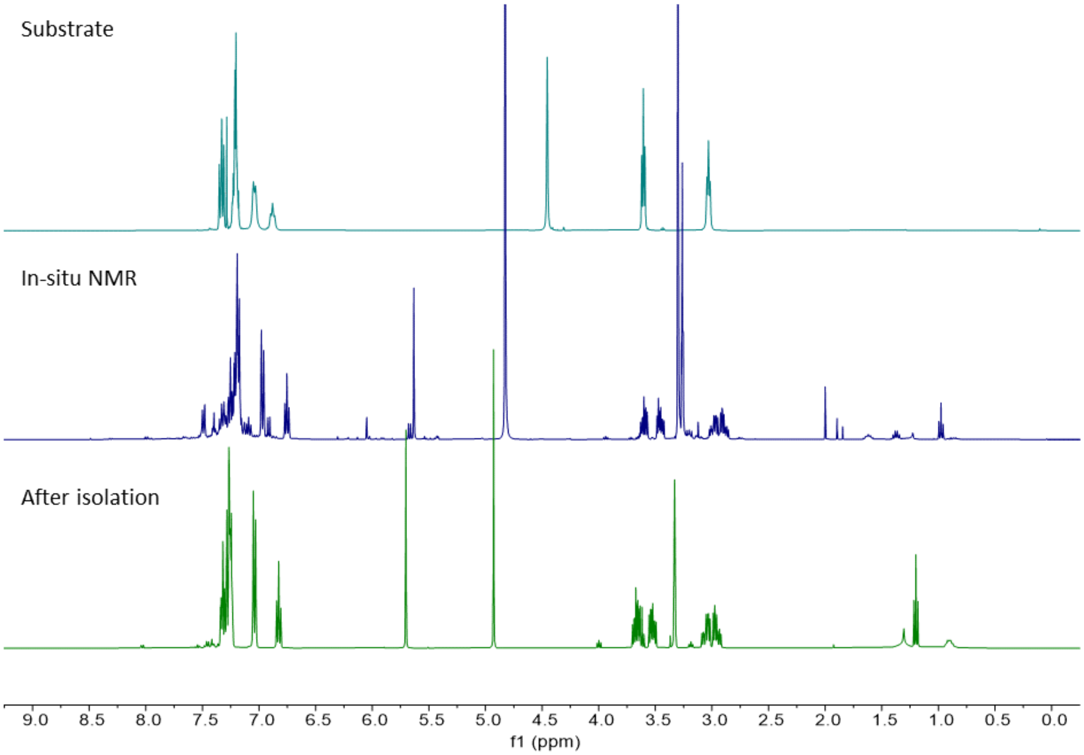

Fig. S16 ^{1}H NMR of the THIQ substrate and product before and after isolation. See literature.[5]

^{1}H NMR of 2a (400 MHz, Methanol-D4)

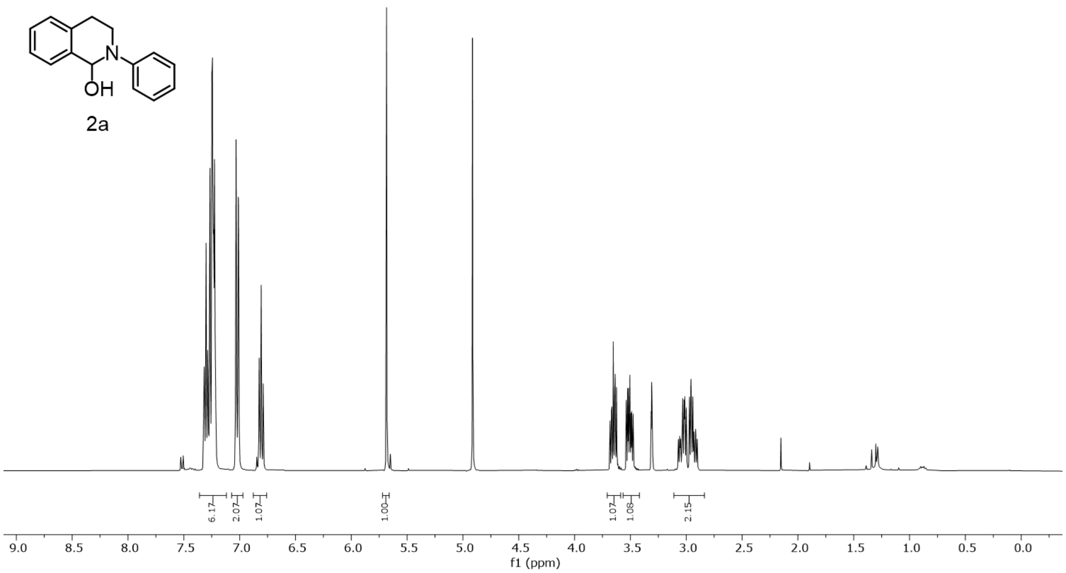

^{1}H NMR (400 MHz, CD_3OD) δ 7.41 – 7.17 (m, 6H), 7.09 – 7.00 (m, 2H), 6.83 (dd, *J* = 7.8, 6.7 Hz, 1H), 5.70 (s, 1H), 3.67 (ddd, *J* = 11.8, 6.8, 5.0 Hz, 1H), 3.53 (ddd, *J* = 11.6, 7.9, 5.0 Hz, 1H), 3.12 – 2.86 (m, 2H). This compound has been reported previously.[5]

^{13}C NMR of 2a (400 MHz, Methanol-D4)

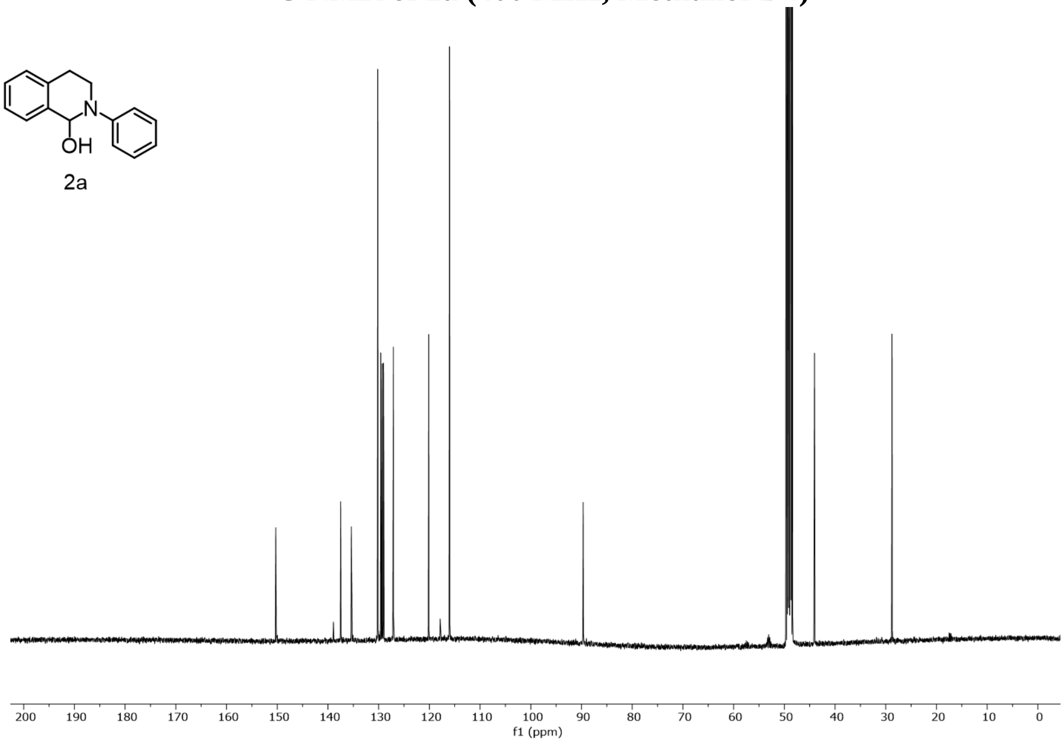

^{13}C NMR (101 MHz, CD_3OD) δ 150.29, 137.49, 135.38, 130.17, 129.57, 129.28, 129.04, 127.10, 120.11, 116.00, 89.66, 44.04, 28.76.

^{1}H NMR of 2b (400 MHz, Methanol-D4)

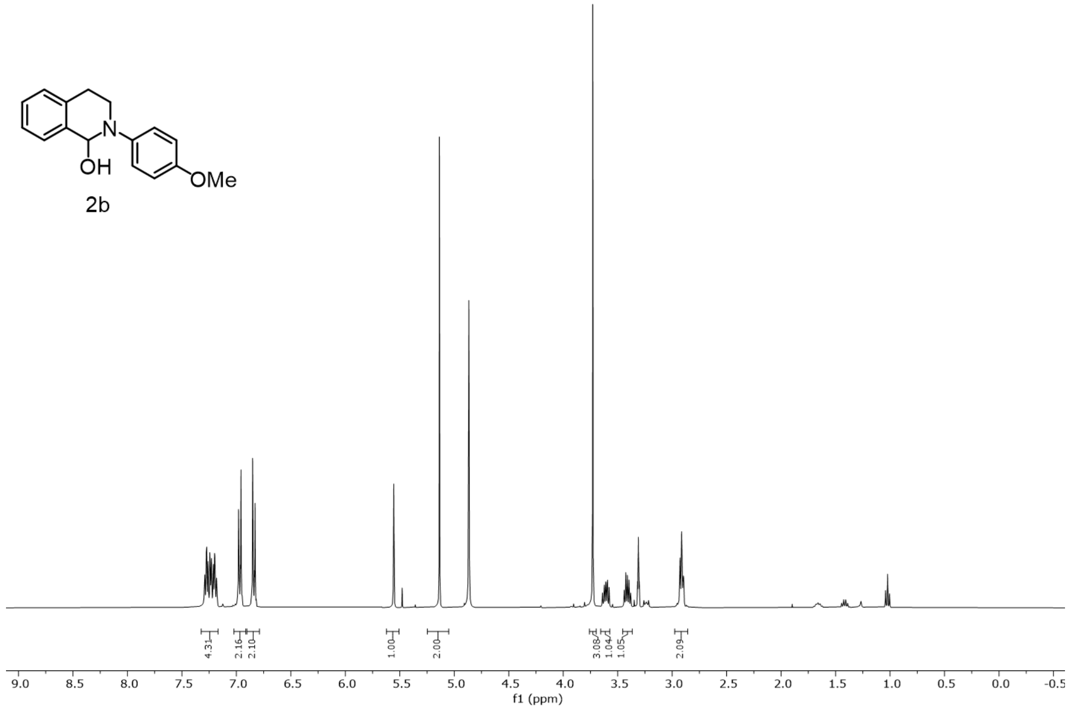

^{1}H NMR (400 MHz, CD_3OD) δ 7.34 – 7.16 (m, 4H), 7.02 – 6.91 (m, 2H), 6.90 – 6.79 (m, 2H), 5.56 (s, 1H), 5.14 (s, CH_2Br_2, 2H), 3.73 (s, 3H), 3.61 (ddd, J = 11.8, 7.1, 5.7 Hz, 1H), 3.41 (ddd, J = 11.8, 5.9 Hz, 1H), 2.98 – 2.86 (m, 2H).

^{13}C NMR of 2b (400 MHz, Methanol-D4)

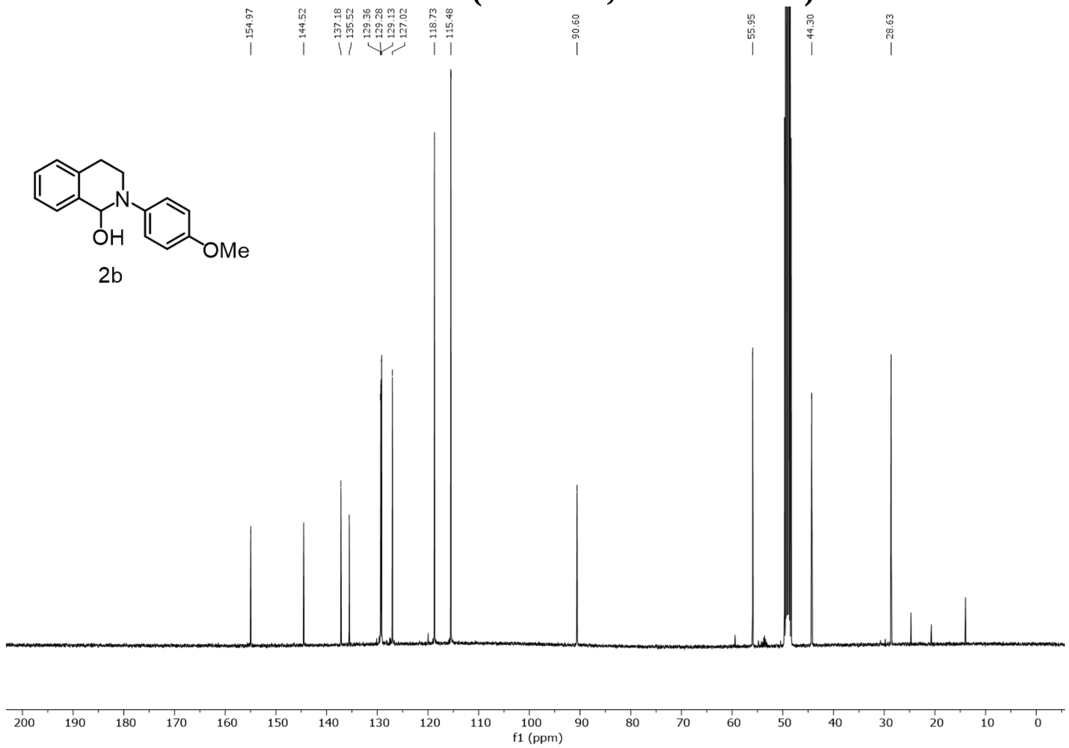

^{13}C NMR (101 MHz, CD_3OD) δ 154.97, 144.52, 137.18, 135.52, 129.36, 129.28, 129.13, 127.02, 118.73, 115.48, 90.60, 55.95, 44.30, 28.63.

^{1}H NMR of 2c (400 MHz, Methanol-D4)

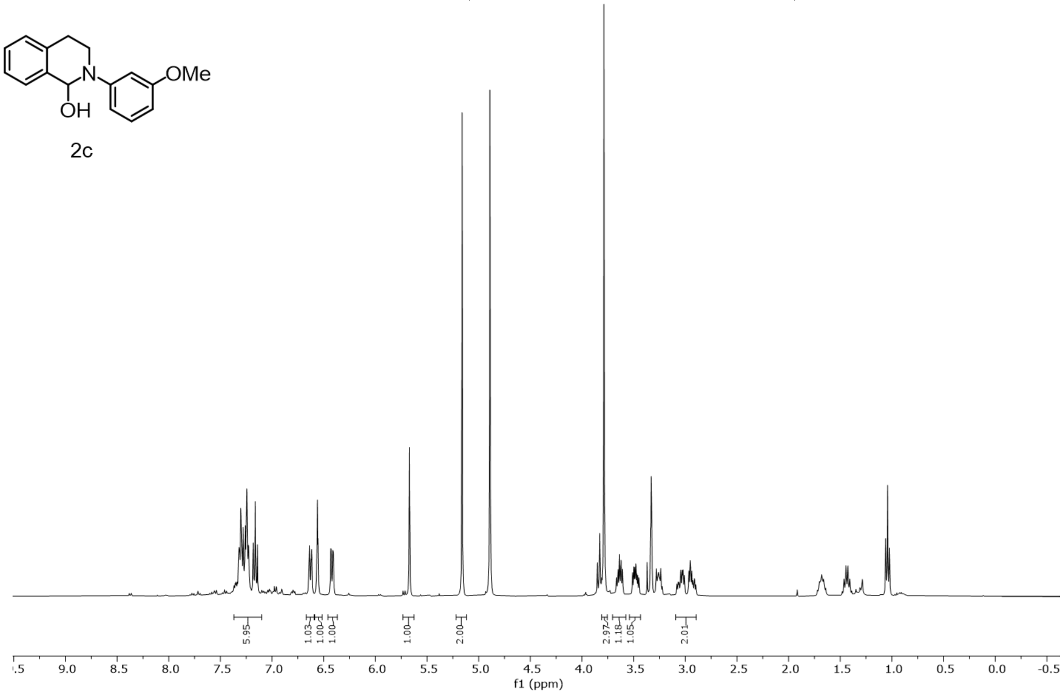

^{1}H NMR (400 MHz, CD_3OD) δ 7.37 – 7.12 (m, 5H), 6.63 (dd, *J* = 8.2, 2.4 Hz, 1H), 6.56 (t, *J* = 2.4 Hz, 1H), 6.46 – 6.37 (m, 1H), 5.67 (s, 1H), 5.16 (s, CH_2Br_2, 2H), 3.79 (s, 3H), 3.64 (ddd, *J* = 11.7, 6.6, 5.1 Hz, 1H), 3.48 (ddd, *J* = 11.6, 8.2, 4.9 Hz, 1H), 3.10 – 2.89 (m, 2H).

^{13}C NMR of 2c (400 MHz, Methanol-D4)

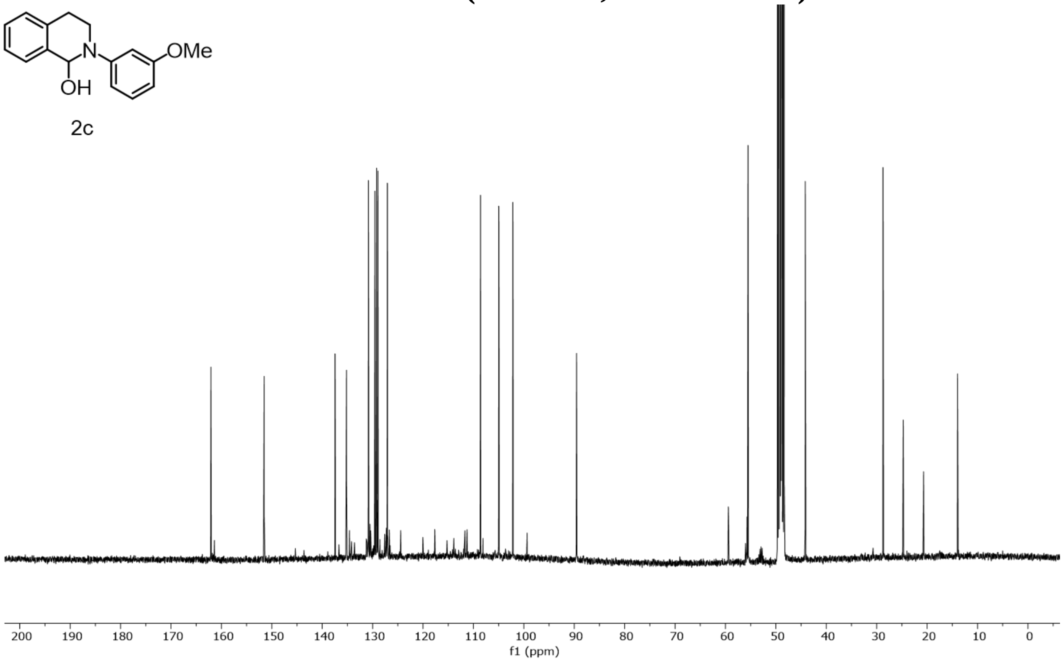

^{13}C NMR (101 MHz, CD_3OD) δ 162.08, 151.52, 137.44, 135.19, 130.83, 129.54, 129.20, 128.93, 127.05, 108.59, 104.96, 102.17, 89.53, 55.51, 44.16, 28.73.

^{1}H NMR of 2d (400 MHz, Methanol-D4)

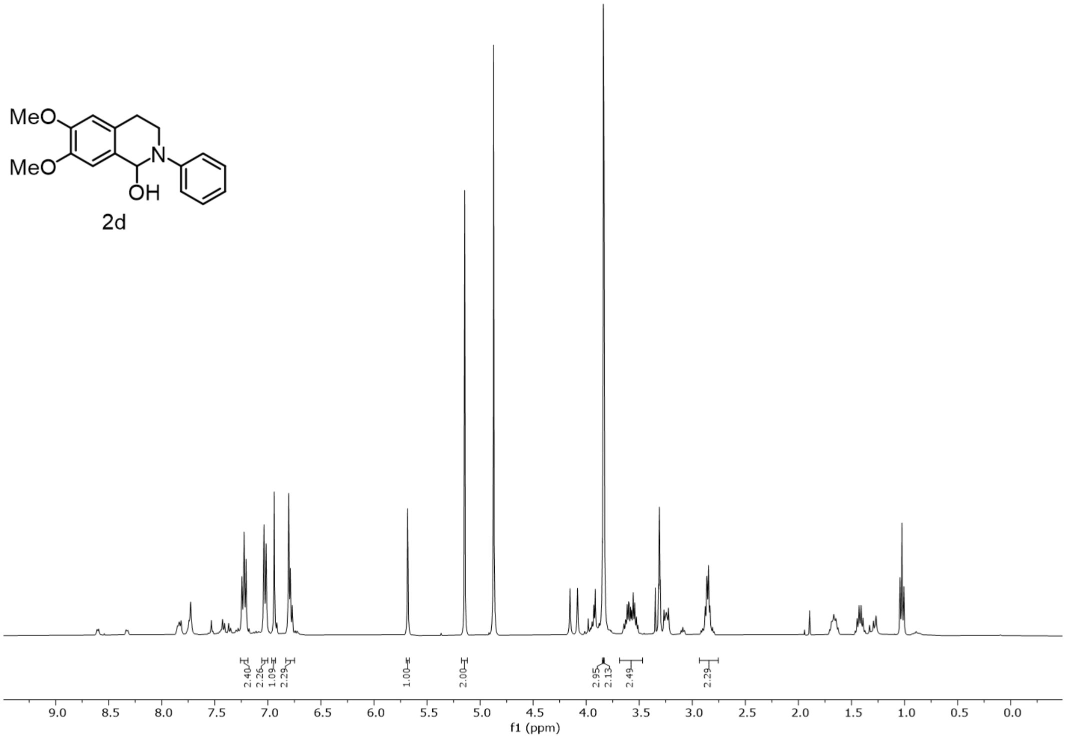

^{1}H NMR (400 MHz, CD_3OD) δ 7.42 – 7.18 (m, 2H), 7.07 – 7.01 (m, 2H), 6.94 (s, 1H), 6.86 – 6.77 (m, 1H), 5.68 (s, 1H), 5.15 (s, CH_2Br_2, 2H), 3.84 (s, 3H), 3.83 (s, 3H), 3.69 – 3.50 (m, 1H), 3.00 – 2.77 (m, 1H).

^{13}C NMR of 2d (400 MHz, Methanol-D4)

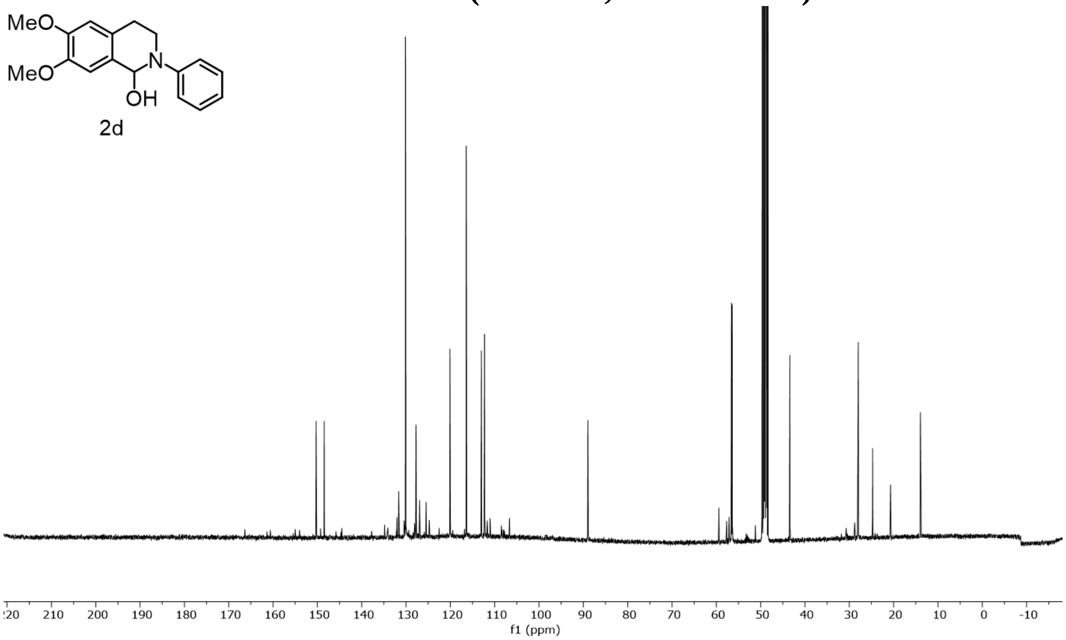

^{13}C NMR (101 MHz, CD_3OD) δ 150.29, 150.23, 148.44, 130.11, 127.74, 120.09, 116.41, 112.99, 112.27, 88.98, 56.58, 56.40, 43.40, 27.99.

^{1}H NMR of 2e (400 MHz, Methanol-D4)

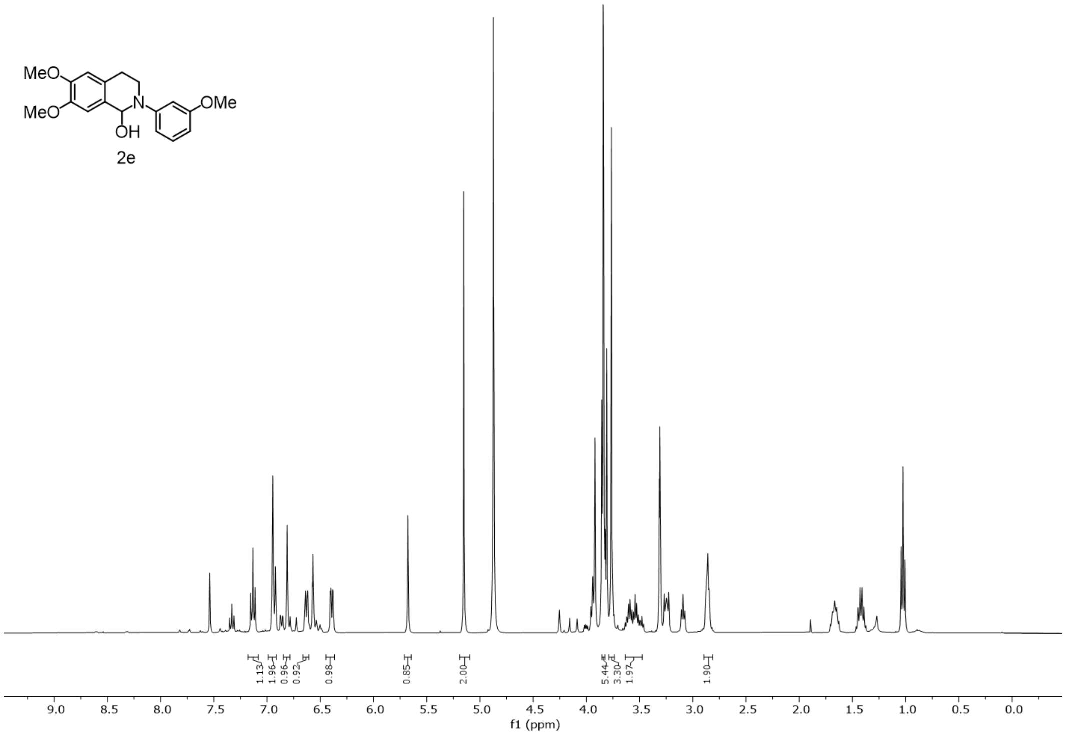

^{1}H NMR (400 MHz, CD_3OD) δ 7.13 (t, *J* = 8.2 Hz, 1H), 6.938 – 6.92 (m, 2H), 6.81 (s, 1H), 6.63 (dd, *J* = 8.4, 2.4 Hz, 1H), 6.39 (dd, *J* = 8.1, 2.4 Hz, 1H), 5.68 (s, 1H), 5.15 (s, CH_2Br_2, 2H), 3.90 – 3.80 (m, 8H), 3.76 (s, 4H), 3.69 – 3.43 (m, 2H), 2.90 – 2.81 (m, 2H).

^{13}C NMR of 2e (400 MHz, Methanol-D4)

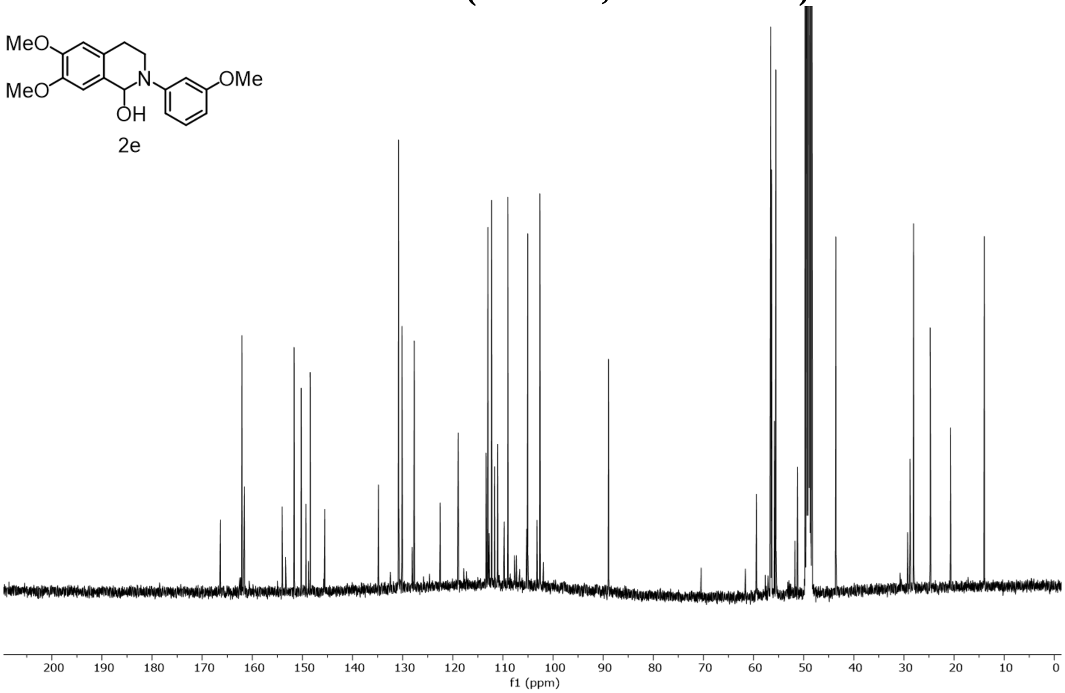

^{13}C NMR (101 MHz, CD_3OD) δ 162.09, 151.63, 150.24, 148.45, 130.82, 130.14, 127.68, 112.99, 112.25, 109.03, 105.04, 102.62, 88.93, 56.58, 55.52, 43.57, 28.06, 13.96.

^{1}H NMR of 2f (400 MHz, Methanol-D4)

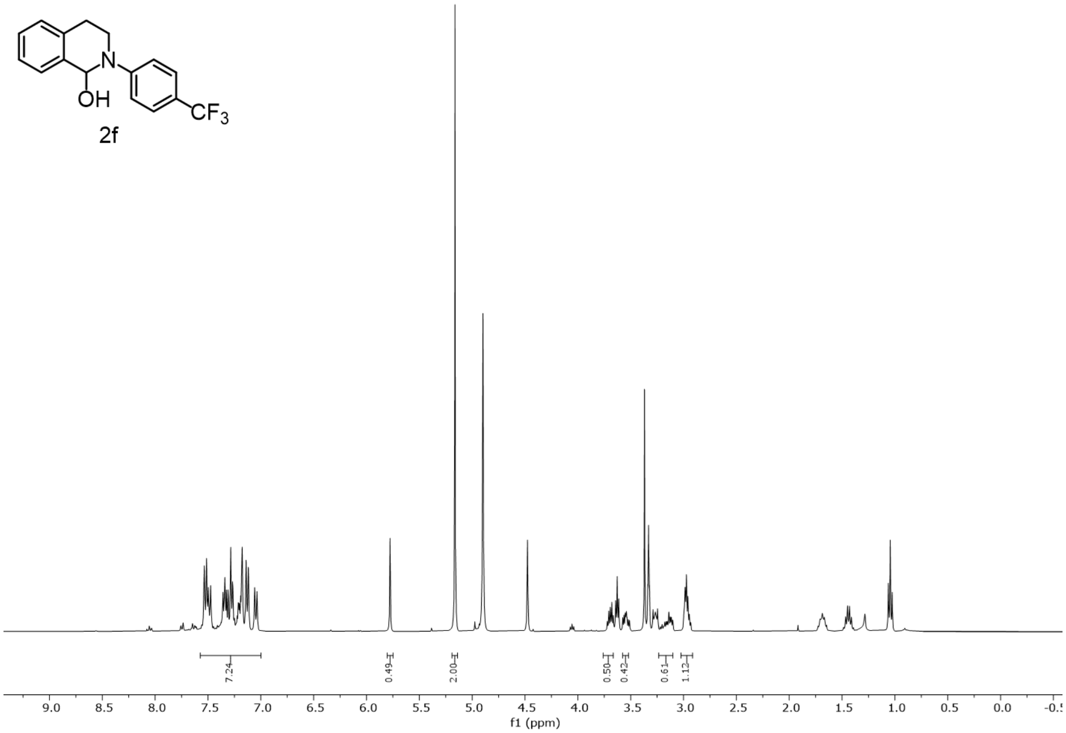

^{1}H NMR (400 MHz, CD_3OD) δ 7.60 – 7.02 (m, 8H), 5.78 (s, 1H), 5.16 (s, CH_2Br_2, 2H), 3.73 – 3.65 (m, 1H), 3.58 – 3.50 (m, 1H), 3.02 – 2.91 (m, 2H).

^{13}C NMR of 2f (400 MHz, Methanol-D4)

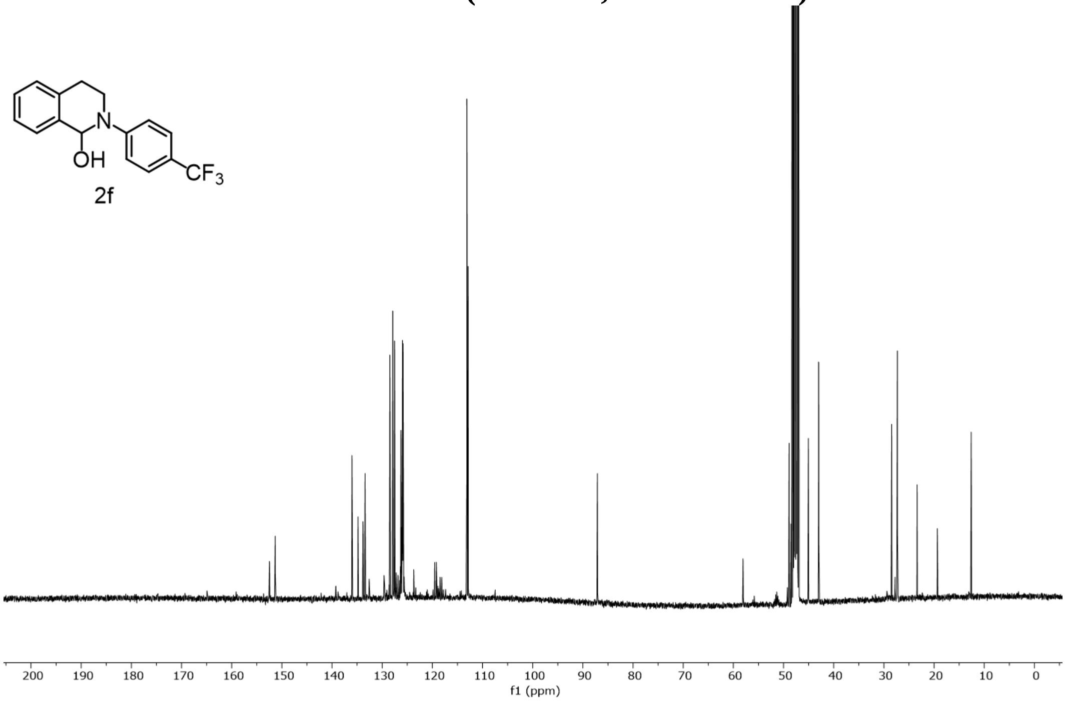

Not analyzed due to contaminations with starting material and/or side products.

Table S1. DCN trace metal analysis by inductively coupled plasma optical emission spectroscopy. DCN was either synthesized directly on float glass and simply peeled off or mechanically scratched off from the FTO substrate. Polystyrene (PS) was used as comparison. The elevated Sn concentration (~0.5 %) in the DCN on FTO sample was caused by tight adhesion of DCN to the FTO, requiring vigorous mechanical scratching with steel tweezers for removal. Most notable contaminations of DCN on glass were ~0.02 % of copper (Cu), zinc (Zn), potassium (K), and calcium (Ca). Sodium (Na) is typically elevated.

	**DCN on glass**	**DCN on FTO**	**PS**
**Analyte**	Concentration units (mg g^{-1})	Concentration units (mg g^{-1})	Concentration units (mg g^{-1})
**Co**	0.007 ± 0.000	0.014 ± 0.000	0.000 ± 0.000
**K**	0.227 ± 0.004	0.327 ± 0.004	0.046 ± 0.000
**Fe**	0.029 ± 0.001	0.560 ± 0.008	0.009 ± 0.000
**Zn**	0.159 ± 0.002	0.330 ± 0.002	0.039 ± 0.001
**Cu**	0.228 ± 0.005	0.364 ± 0.005	0.045 ± 0.001
**Ti**	0.002 ± 0.000	0.002 ± 0.000	0.000 ± 0.000
**Ni**	0.013 ± 0.000	0.148 ± 0.002	0.004 ± 0.000
**Na**	2.340 ± 0.050	3.320 ± 0.059	0.350 ± 0.002
**Mg**	0.025 ± 0.000	0.035 ± 0.001	0.005 ± 0.000
**Ca**	0.268 ± 0.005	0.414 ± 0.013	0.065 ± 0.001
**Al**	0.037 ± 0.001	0.030 ± 0.001	0.004 ± 0.000
**Sn**	0.034 ± 0.001	4.950 ± 0.070	0.020 ± 0.000

Supplementary Discussion 3

Given the relatively low mass of the DCN film (presumably few hundreds of micrograms), we estimated the Cu content in respect to the substrate in the reaction mixture to be ~0.005 mol. %, by taking into account (1) the volume of a carbon nitride layer (L x W x H) of 20 mm x 10 mm x 1.8 µm with a carbon nitride gravimetric density of ~2 g cm^{-3},[7] (2) the metal content determined by ICP in the reaction mixture, and (3) the loading of N-aryltretrahydroisoquinoline (50 µmol). This amount of metal is 200x lower compared to, for example, the amount of homogeneous copper dinuclear complex used in a similar reaction.[5] It is unlikely that the hydroxylation of N-aryltetrahydroisoquinoline exclusively occurs due to such a low catalytic quantity of metal. However, at this point we cannot fully exclude the role of trace metal impurities as catalysts in the studied reactions.

Table S2. Current density (μA cm^{-2}) of DCN electrodes from different synthesis conditions (polymer type, polymer concentration, and synthesis temperature) at +1.23 V *vs*. RHE with 0.1 M Na_2SO_4 in water.

Polymer/ concentration (% w/w)	S-LEC	PS	PVP	PEG		S-LEC	PS	PVP	PEG		S-LEC	PS	PVP	PEG
**25**	84	40	55	20		123	198	59	237		466	575	6	107
**35**	47	32	3	26		471	641	15	14		588	225	13	110
**50**	20	27	5	21		25	592	37	89		664	620	8	30
**60**	200	20	4	666		67	910	5	588		434	278	15	58
	550 °C					560 °C					570 °C			

Table S3. Current density (μA cm^{-2}) of DCN electrodes from different synthesis conditions (polymer type, polymer concentration, and synthesis temperature) at +0.22 V *vs.* Fc+/Fc using 0.1 M $LiClO_4$ in methanol.

Polymer/ concentration (% w/w)	S-LEC	PS	PVP	PEG		S-LEC	PS	PVP	PEG		S-LEC	PS	PVP	PEG
**25**	52	19	13	9		49	322	15	89		153	178	4	49
**35**	32	12	5	21		105	432	9	17		162	230	14	29
**50**	15	15	6	11		19	578	7	20		119	372	7	16
**60**	86	6	5	111		47	173	2	119		167	91	9	37
	550 °C					560 °C					570 °C			

References

1. Zhang J, *et al.* Laser-driven growth of structurally defined transition metal oxide nanocrystals on carbon nitride photoelectrodes in milliseconds. *Nat Commun* **12**, 3224 (2021).
2. Condie AG, González-Gómez JC, Stephenson CRJ. Visible-Light Photoredox Catalysis: Aza-Henry Reactions via C−H Functionalization. *J Am Chem Soc* **132**, 1464-1465 (2010**)**
3. Möhlmann L, *et al.* Carbon Nitride-Catalyzed Photoredox C-C Bond Formation with N-Aryltetrahydroisoquinolines. *Adv Synth Catal* **354**, 1909-1913 (2012)
4. Xie WX, *et al.* Diethyl Phosphite Promoted Electrochemical Oxidation of Tetrahydroisoquinolines to 3,4-Dihydroisoquinolin-1(2H)-ones. *Synlett* **30**, 2077-2080 (2019)
5. Liu Y, *et al.* Reactions Catalysed by a Binuclear Copper Complex: Relay Aerobic Oxidation of N-Aryl Tetrahydroisoquinolines to Dihydroisoquinolones with a Vitamin B1 Analogue. *Chem Eur J* **23**, 3062 (2017)
6. Dighe U, *et al.* A photochemical dehydrogenative strategy for aniline synthesis. *Nature* **584**, 75–81 (2020)
7. https://www.americanelements.com/graphitic-carbon-nitride-143334-20-7, accessed October 9, 2023
